# Osteosarcopenia—The Role of Dual-Energy X-ray Absorptiometry (DXA) in Diagnostics

**DOI:** 10.3390/jcm11092522

**Published:** 2022-04-30

**Authors:** Aleksandra Gonera-Furman, Marek Bolanowski, Diana Jędrzejuk

**Affiliations:** 1 Department of Endocrinology, Diabetes and Isotope Therapy, Jan Mikulicz-Radecki University Clinical Hospital, 50-556 Wroclaw, Poland; agonerafurman@gmail.com; 2 Department of Endocrinology, Diabetes and Isotope Therapy, Wroclaw Medical University, 50-367 Wroclaw, Poland; marek.bolanowski@umw.edu.pl

**Keywords:** osteosarcopenia, DXA, bone density, bone quality, fracture risk, body composition

## Abstract

Osteoporosis and sarcopenia lead to increased mortality, but their early diagnosis allows preventive measures and treatment to be implemented. The dual-energy X-ray absorptiometry (DXA) method enables the assessment of both bone mineral density (BMD) and bone quality based on the trabecular bone score (TBS), the Bone Strain Index (BSI), hip structure analysis (HSA), and comprehensive hip axis length (HAL). The main complications of osteoporosis are fractures, and a BMD value or T-score together with TBS can be also applied in fracture risk calculation using the Fracture Risk Assessment Tool (FRAX). In recent years, the interest in sarcopenia has increased. There are many methods for assessing the quality, quantity and function of muscles. Total body DXA provides information not only about the BMD of the whole skeleton or the amount of lean tissue (identified as fat-free mass), but also about the amount and distribution of adipose tissue. Some parameters obtained from DXA measurements related to muscle and/or fat mass are used in the assessment of osteosarcopenia. The following article presents a wide range of possibilities for the use of the DXA method in the diagnosis of osteosarcopenia because DXA is a useful technique for the diagnosis of bone density and body composition together.

## 1. Introduction

Osteosarcopenia is a recently proposed name for a syndrome involving the co-occurrence of osteoporosis and sarcopenia. The consequences of osteosarcopenia include an increased risk of falls and fractures, leading to a significant public health burden [1]. The diagnostic criteria used for osteoporosis and sarcopenia were established separately. 

The clinical definition of osteoporosis states that it is a systemic skeletal disease characterized by a low bone mass and the microarchitectural deterioration of bone tissue, with a consequent increase in bone fragility and susceptibility to fracture [2]. Therefore, bone quantity, bone microarchitecture and bone geometry constitute three equal elements that should be taken into consideration in each case. Dual-energy X-ray absorptiometry (DXA) is a helpful technique for assessing bone quantity, bone quality and bone geometry in order to quantify fracture risk. DXA allows for the comprehensive quantification of bone mineral density (BMD), trabecular bone score (TBS), Bone Strain Index (BSI), hip structure analysis (HSA) and hip axis length (HAL). Apart from bone mineral density (BMD), other practical tools complete the diagnosis, such as the TBS and FRAX.

Sarcopenia is a progressive and generalized skeletal muscle disorder associated with an increased likelihood of adverse outcomes including falls (which may result in fractures), physical disability and a higher rate of mortality [3,4]. According to the definition established in 2018 by the European Working Group on Sarcopenia in Older People (EWGSOP), the risk of sarcopenia is increased when low muscle strength is detected. The diagnosis is confirmed upon recording the presence of low muscle quantity or quality. When low muscle strength, low muscle quantity/quality and low physical performance are detected simultaneously, sarcopenia is regarded as severe. The EWGSOP proposed a diagnostic pathway, including screening for the risk factors for sarcopenia and assessing skeletal muscle strength, mass and quality [5]. Although functional tests are essential for evaluating muscle strength and physical performance, imaging techniques (especially DXA) are commonly used to measure muscle quantity.

In this review, we would like to present a clinical approach to osteosarcopenia focusing on the role of densitometric tools in diagnostic procedures. This paper is a summary of recommendations regarding osteoporosis and sarcopenia that link them to clinical applications.

## 2. The Role of DXA in Osteosarcopenia

### 2.1. Advantages of DXA

DXA is a noninvasive, nonexpensive and widely available technique that exposes patients to a low dose of radiation and requires a very short scanning time (depending on localization, at least 1–3 min) [6,7,8]. The effective dose for anterior–posterior (AP) spine DXA examination is approx. 10 μSv (for reference, the effective dose for lateral spinal X-ray is approx. 600 μSv) [9]. This is the standard used in assessing bone mineral density (BMD). It allows for the evaluation of the lumbar spine, proximal femur, radius and total body BMD [10,11]. Moreover, DXA is currently favoured for measuring body composition using a total body scan [12,13,14,15]. It has been published that performing a total body DXA scan with the rather new generation of densitometers exposes patients to about 4–5 μSv, which is lower than the natural background dose (6.7 μSv per day) [16]. 

### 2.2. Technical Aspects of DXA

Under the DXA method, the X-ray tube emits beams of radiation with two different energies that are attenuated based on both the density and thickness of anatomical structures and tissues, as well as on the intensity of emitted energy [17]. The principle of X-ray attenuation is that the higher the photon energy is, the lower the attenuation will be. On the contrary, an X-ray beam with a lower energy will be more attenuated by tissues, although X-ray beam attenuation is high in high-density tissues (bone) [18]. DXA produces a so-called R-value, which is the ratio between the attenuation coefficients at the two energy levels. R-value is constant for bone and fat in all individuals, although it varies for soft tissue as it depends on the patient’s composition. If a subject has a high fat percentage, their R-value will be lower than that of a subject with a high lean mass percentage [19]. Using the DXA method allows for distinguishing between three different compartments based on their X-ray attenuation properties: bone mineral content (BMC); lipids (triglycerides, phospholipid membranes, organ, marrow and subcutaneous adipose), which is the so-called fat mass (FM); and lipid-free soft tissue, which is the lean mass (LM) [17]. Lipid-free soft tissue (lean soft tissue mass—LSTM) represents bone-free, fat-free soft tissue mass and is the sum of body water, protein, glycogen and soft tissue mineral mass. It is very important to know that the value of fat-free mass (FFM) is the sum of all non-body lipids (FFM = LSTM + BMC) [19] and that soft tissue mass (STM) is the sum of lean soft tissue and FMs (STM = FM + LSTM) [20].

Based on this, the evaluation of DXA is typically considered a three-compartment model, although this technique cannot directly estimate all three different components. About 40–45% of the total area of a DXA scan is composed of bone. In this area, DXA can only differentiate between bone and the global amount of soft tissue, which includes both FM and LM. Pixels that are located adjacent to bone, which contain soft tissue only, are used to calculate the exact FM and LM quantities [20]. Depending on the source of X-ray emission, we distinguish densitometers with a pencil beam from densitometers with a fan beam (currently the most widely represented). A characteristic of pencil-beam densitometers is that they emit a single, rectilinear, highly collimated beam of X-rays coupled with a single detector. On the contrary, fan-beam technology uses a fan-shaped beam that is coupled with multiple detectors, allowing shorter scan times with better image resolutions [18].

### 2.3. Bone

#### 2.3.1. Bone Mineral Density

In the absence of low-trauma fractures, BMD assessed using DXA is the gold standard for diagnosing osteoporosis. Moreover, in some countries (e.g., Poland), reimbursed antiresorptive medications can be prescribed only if such diagnosis is confirmed using a DXA test. BMD is described as a T-score or Z-score, which are units of standard deviation. The T-score is the number of standard deviations above or below the mean reference value for young healthy adults, and the Z-score is the number of standard deviations above or below the mean reference value for age and gender. T-scores are calculated by taking the difference between a patient’s measured BMD and the mean BMD in healthy young adults, matched for gender and ethnic group. T-score = (measured BMD−young adult mean BMD)/young adult population SD [21]. Z-scores are similar to T-scores except that instead of comparing the patient’s BMD with the young adult mean, it is compared with the mean BMD expected for the patient’s peers (e.g., for a healthy normal subject matched for age, gender and ethnic group). Z-score = (measured BMD) − (age-matched mean BMD)/age-matched population SD [22].

The WHO reference standard (1994) is a T-score of −2.5 or less at the femoral neck [2]. However, according to the main American and European guidelines, the preferred measurement sites include the total hip, femoral neck and/or lumbar spine (L1 to L4) [10,11]. In the case of hyperparathyroidism, severe obesity, pregnancy (in special cases) and when the hip or spine cannot be measured or the measurements cannot be interpreted, it is recommended to use 33% of the radius (1/3 radius) [10,11,12].

These criteria refer to postmenopausal women and men aged 50 and older [12]. This is the arbitrarily agreed-upon age limit related to decreases in sex hormone concentration (especially estrogen) and its beneficial impacts on bones. Younger patients (women over 20 until menopause and men between 20 and 50 years old) must have their Z-score calculated: a Z-score of −2.0 and lower is defined as ”below the expected range for [this] age” and a Z-score above −2.0 is regarded to be ”within the expected range for [this] age.” For women without prior low-trauma fracture, BMD is the single best predictor of osteoporotic fracture risk [10].

There have been some data on the usefulness of peripheral bone density measurements (e.g., wrist, mid-thigh) in the diagnosis of osteoporosis [23,24], although diagnostic densitometric criteria apply only to the axial measurements and the distal 1/3 of the radius. These locations are the most common sites of osteoporotic fractures, and hip and vertebral fractures cause significant disability and are associated with a high rate of mortality. Distal radius fractures known as “loco typico” are one of the most frequent fractures in the population (including low-energy osteoporotic fractures) and mainly affect women in early menopause. Fractures of the distal radius more often lead to complex regional pain syndrome (CRPS) than to disability [25]. Other peripheral sites can be used to identify patients with an increased risk of fracture [10,12].

#### 2.3.2. Bone Quality

BMD is a quantitative method of bone assessment. Due to two-dimensional imaging and limitations of the measurement method (pencil beam and fan beam scan), DXA results can be inadequate. Pencil-beam measurements are more accurate and emit lower amounts of radiation but take longer, while fan beam scanning is faster but produces more artifacts [17]. BMD can be overestimated in patients with degenerative changes in their spine and overlying structures, such as aortic calcification. It does not differentiate trabecular BMD from cortical BMD, which can result in the misdiagnosis of pathological changes caused by, for example, glucocorticoid therapy or rheumatoid disease. However, DXA-based methods such as the trabecular bone score (TBS), vertebral fracture assessment (VFA), hip structural analysis (HSA) and Bone Strain Index (BSI) allow for assessing bone quality and detecting fractures [26,27,28,29,30].

In fact, only TBS values and, indirectly, VFA results (the presence or absence of vertebral fractures) are taken into account with the fracture risk assessment tools (e.g., FRAX). 

TBS provides information on bone texture. The TBS value is calculated directly from the DXA scan data concerning the lumbar spine area (L1–L4). It is based on the analysis of the optical density texture of a 2D bone density image, which is a projection of a 3D structure. A large number of pixels with a low amplitude variation indicates a dense trabecular structure, while a small number of pixels with a high amplitude variation is related to a sparse, osteoporotic bone architecture [31]. A TBS value higher than 1.350 is considered a normal microarchitecture, between 1.200 and 1.350 is regarded as partially degraded and values lower than 1.200 indicate degraded microarchitecture [32,33]. According to the International Society for Clinical Densitometry (ISCD), TBS is associated with vertebral, hip and major osteoporotic fracture risk in postmenopausal women and with hip fracture risk in men over the age of 50 [12]. The indirect assessment of bone structure with TBS can provide beneficial information in cases of secondary osteoporosis much more accurately than BMD. In the case of, for example, diabetes mellitus, parathyroid dysfunctions, renal failure, chronic arthritis and glucocorticoid-induced osteoporosis, TBS values were found to reveal additional microarchitectural changes when the BMD values were comparable [31,34,35]. However, the Official Position of the ISCD indicates diabetes mellitus type II in postmenopausal women to be a condition in which the TBS is associated with major osteoporotic fracture risk [12,36]. This is why further research is necessary. Routinely, TBS values are calculated from DXA scans of the lumbar spine. There are limited data on the use of the TBS tool in different areas, although the role of TBS (in the lumbar protocol) in the knee area in individuals with spinal cord injury has been investigated [37]. 

Bone quality can also be evaluated based on the detection of vertebral and femoral fractures. VFA by DXA scanning is typically performed to diagnose asymptomatic vertebral fractures in thoracic and lumbar locations, which is important for: (1) detecting subclinical vertebral fractures, which may modify risk category and thus commencement or type and duration of therapy, depending on age and local criteria for intervention and (2) providing baseline assessment, based on which later incident vertebral fractures can be discriminated from prevalent fractures, which is critical to optimal treatment monitoring [26]. Compared with standard spine radiographs, the correlation between detection of moderate and severe vertebral fractures is good, with a smaller dose of ionizing radiation (from 2 to 50 μSv for VFA vs. 600 μSv for lumbar spine radiograph) [38]. This method is limited by the necessity of performing a DXA scan in projections other than a standard AP spine DXA exam (in scanners without a rotating c-arm, the patient should lie on their side, which can be problematic, especially in the case of elderly people with severe back pain, significant scoliosis or disability). VFA has a good sensitivity and excellent specificity compared with spinal radiographs for moderate and severe vertebral fractures (>25% vertebral height loss). In cases where mild fractures (<25% height loss) are suspected, confirmation with spinal radiography should be considered. According to the ISCD, VFA is recommended for patients with at least osteopenia and when one or more of the following elements is present: the patient is above 70 years of age in the case of women and above 80 in the case of men, their height loss is over 4 cm or there has been a self-reported but undocumented vertebral fracture or glucocorticoid intake [12,39].

A hip structure analysis (HSA) provides information on hip geometry and the mechanical properties of the femur. It measures cross-sectional geometry in three different regions of interest: the narrowest part of the femur neck, the intertrochanteric region and the femoral shaft. For each location, the distribution of the bone mass is computed, while mechanical properties are derived from the femur geometry. The most important parameters include the cross-sectional area (CSA), indicative of the bone surface area in the cross-section, and the cross-sectional moment of inertia (CSMI), which describes how the bone mass is distributed around the femoral axis. The higher the CSA and CSMI are, the better the bone resistance to axial compression and bending will be, respectively. However, the interpretation of the results is problematic, and there is insufficient evidence in clinical practice regarding fracture prediction [27]. Therefore, the ISCD does not recommend HSA parameters for assessing hip fracture risk [12].

Recently, a new DXA-derived index referred to as the Bone Strain Index (BSI) has been proposed to evaluate the capacity of bone to withstand an applied load. The BSI is calculated taking into account the patient’s height and weight, and it represents the average equivalent strain inside the bone. The BSI can be a predictor of elastic and plastic changes in the mechanical response of the bone. As a consequence, the BSI can be applied to predict a possible location of fracture within the vertebrae or femur. The higher the BSI is, the more fragile the bone will be. In clinical studies, the BSI appears to be useful in identifying osteoporotic patients who are particularly prone to fragility fractures and characterizing young patients affected by secondary osteoporosis [27,40]. However, currently, the BSI is considered only as an additional tool; the international guidelines do not mention it in any recommendations.

#### 2.3.3. Fracture Risk

Clinically, osteoporosis is diagnosed after detecting a fragility fracture, a low-trauma fracture sustained from a force similar to a fall from a standing or lower position that would not have occurred in a healthy bone (excepting fractures of the skull, face, fingers and toes). Major sites of osteoporotic fractures include the hip, spine, distal radius and proximal humerus. Low-trauma fractures may also occur in the pelvis, sacrum, ribs, distal femur, distal humerus and ankle (minor sites). An osteoporotic fracture is not always associated with a low bone density equivalent to osteoporosis. Prior history of fractures in any of the aforementioned sites is an important predictor of future incidents, particularly in adults over the age of 55 [41] or even younger [42].

The detection of a previously undiagnosed case (e.g., asymptomatic vertebral fractures found by means of VFA) may change the diagnostic classification, assessment of fracture risk and treatment plan [43]. Fractures can be caused by many factors. The most important osteoporosis risk determinants are included in Table 1.

Significant, but often overlooked, nonskeletal determinants of fractures involve susceptibility to falls, usually associated with comorbidities (especially neurological diseases), polypharmacy (including antidepressants, antihypertensives and nonsteroidal anti-inflammatory and antipsychotic drugs) or other factors which are not taken into consideration as a standard. Interestingly, even self-reported risk of fracture was proved to be an independent predictor [49,50]. A universal algorithm for fracture risk evaluation is currently under development. Today, there are three main risk assessment tools: FRAX, the Garvan fracture risk calculator and QFracture. FRAX, designed by John Kanis, is an international algorithm based on a series of meta-analyses of data derived from 12 independent studies. It calculates the 10-year probability of major fractures (of the hip, spine, humerus and wrist) and the 10-year probability of hip fracture. However, although the main risk factors are taken into account in the calculation, the susceptibility to falls is not included. The Garvan calculator is based on a much smaller study group (data from a single Australian study), but it includes the patient’s history of falls and their number of previous fragility fractures. Furthermore, it details the risk of more fracture sites. The QFracture tool is based on a British prospective open cohort study and is not calibrated to the epidemiology of other countries [51]. Due to the versatility and international character of FRAX, it is commonly recommended in guidelines [7,11,52]. FRAX is a part of the definition of osteoporosis proposed by the National Bone Health Alliance Working Group, according to which a diagnosis of osteoporosis requires a BMD-based T-score of ≤−2.5 at the hip (total hip or femoral neck) or lumbar spine, a low trauma fracture or a FRAX score at the intervention thresholds (in Poland, ≥10% for a major osteoporotic fracture). This approach increases the prevalence of osteoporosis but also improves the chance to prevent fragility fractures [52,53]. To enhance fracture risk prediction, femoral neck BMD and lumbar spine TBS can be taken into consideration as well. TBS is a significant and independent predictor of fractures [54]. It is useful in the assessment of fracture risk in the case of certain causes of secondary osteoporosis (e.g., hyperparathyroidism, diabetes, glucocorticoid-induced osteoporosis) [11]. FRAX models have been developed by studying population-based cohorts from Europe, North America, Asia and Australia, which is why the same risk factors and BMD values produce different fracture risks depending on the country. The extant findings show how heterogenous osteoporosis is and how many factors need to be considered when diagnosing it.

HAL is the next interesting value considered to be proportionally associated with fracture risk. It is defined as the distance from the inner pelvic brim to the greater trochanter (IT). HAL predicts hip fracture in postmenopausal women but not in men. In addition, the evidence in women shows that fracture risk is independent of BMD, suggesting that the measurement may be clinically useful in postmenopausal women. In the data from the Manitoba database, HAL predicts hip fracture even when fracture risk assessment tool (FRAX) and BMD are adjusted for. These findings suggest that it may be possible to develop an algorithm for adjusting FRAX probability with HAL measurements that could be used in clinical practice [55].

### 2.4. Body Composition (BC)—The Role in the Diagnosis of Sarcopenia

DXA provides whole-body and regional estimates of three different values based on their specific X-ray attenuation properties: bone mineral content (BMC), fat mass (FM) and lean body mass (LM). Although cross-sectional techniques (CT, MRI) are considered the gold standard in the assessment of body composition, DXA is capable of evaluating LM and FM with relatively high accuracy. One technical limitation is that DXA measurements can be influenced by the hydration status of the patient, which can lead to the overestimation of LM and underestimation of FM. However, owing its safety and availability and practically no contraindications, there is a consensus that DXA should be considered the method of reference for the assessment of BC in clinical practice [5,6,56,57,58].

#### 2.4.1. Muscle Mass

When diagnosing sarcopenia, DXA allows for evaluating regional and total body muscle mass. The most important indices in muscle assessment include appendicular lean mass (ALM) and its height²-adjusted ALM index (ALMI). The lower cut-off points proposed by the EWGSOP in 2018 are: ALMI < 7.0 kg/m^2^ for men and ALMI < 5.5 kg/m^2^ for women. The absolute values of ALM are: <20 kg for men and <15 kg for women [5]. Cut-off points may change depending on, among other things, nationality, gender, age and coexistence of chronic conditions. It should be highlighted that these indices should not be used alone to diagnose sarcopenia; both low muscle quantity and quality must be detected to confirm such diagnosis. Therefore, low muscle function (muscle strength measured by, e.g., grip strength or performance assessed by, e.g., the Timed Up and Go test or a 400 m walking test) should be noted [5,57]. Associations such as the EWGSOP or the Asian Working Group for Sarcopenia (AWGS) recommend including ALMI as a muscle mass index together with muscle strength/physical performance indices in diagnosing sarcopenia [5,59]. Low lean mass was revealed to be highly associated with increased mortality in patients regardless of their condition (i.e., cardiovascular diseases, cancer, liver disease or simply elderly age) and measurement modalities [60]. However, these recommendations are inconsistent with statements from the Sarcopenia Definition and Outcomes Consortium, which claims that ALMI is not a good predictor of adverse health-related outcomes such as mobility limitations, falls, disability and mortality and that they thus should not be included in the definition of sarcopenia [61]. Moreover, another study revealed that ALMI is a poor predictor of incident fractures after adjustment for femoral neck BMD [62]. Perhaps the combination of DXA derivatives with other muscle parameters would prove to be a better diagnostic tool, but it is necessary to conduct further research in this regard—e.g., ALMI with myokine concentration [63], ALMI with lower-extremity muscle strength tests [64] or ALM with bioimpedance spectroscopy (BIS) [65].

#### 2.4.2. Fat Tissue

In addition to muscle mass measurement, DXA allows for estimating total and regional body fat. Adipose indices obtained using whole-body DXA provide data on fat distribution. The most commonly used are the fat mass index (FMI), the android/gynoid ratio (AG), visceral adipose tissue (VAT) and the trunk/legs % fat and trunk/limb fat mass ratios. FMI, calculated as the total body fat mass adjusted by height^2^, is the densitometric equivalent of BMI, but its usefulness is still to be discussed [66,67]. It can be used to define obesity. Table 2 shows the cut-off points proposed as part of the National Health and Nutrition Examination Survey (NHANES). The android/gynoid ratio (AG) is similar to the anthropometric measurement of the waist-to-hip ratio [68]. The higher the fat tissue content in the android deposit, and the less the fat tissue content in the gynoid deposit, the increased cardiovascular risk [69,70,71]. The visceral adipose tissue (VAT) is estimated by subtracting the amount of subcutaneous fat (SAT) in the android region from the total fat mass in this region [6]. It is considered to be a better predictor of cardiovascular risk than AG [72,73,74]. The cut-off points of VAT and AG are still being discussed [75,76]. The assessment of trunk/limb fat mass using the trunk/leg and trunk/limb fat mass ratios is useful in the evaluation of lipodystrophy or lipoatrophy [6,56]. The evaluation of fat indices gives additional information about cardiovascular and metabolic risk [77,78]. Reduced lean body mass in the context of excess adiposity is referred to as sarcopenic obesity [79]. This is a distinct condition that exacerbates sarcopenia and increases the risk of physical disability and mortality [80].

All the muscle and fat parameters mentioned above can be used in various medical branches, e.g., cardiology, oncology or nephrology, to adjust the risk associated with the disease or medical procedure and, therefore, to individualize treatment strategies.

### 2.5. Limitations

This paper aimed to summarize the recent knowledge obtained about different possibilities for the use of DXA in the diagnosis of osteoporosis and sarcopenia. The potential beneficiaries of this article include not only clinicians involved in osteosarcopenia management but also, and maybe most importantly, general practitioners and doctors with other specialties. In our opinion, knowledge about the DXA technique and its benefits is insufficient. We are aware of the limitations of this article. It includes only general information about particular parameters, without providing technical details or clinical examples. We did not compare DXA with other imaging techniques. The use of DXA in the assessment of body composition has been presented mainly in relation to sarcopenia. However, it should be emphasized that research on the role of body composition in the assessment of disease risk and morbidity is currently underway. Regardless of these facts, we hope it is a valuable source of knowledge.

## 3. Conclusions

DXA is the most common technique used in the assessment of both components of osteosarcopenia. It allows for evaluating fracture risk by measuring mineral density, analyzing its microarchitecture and resistance and detecting asymptomatic fractures. The data obtained are important variables in the evaluation of fracture risk. In fact, it is the most clinically usable method for osteoporosis assessment. Whole-body DXA imaging provides information on the content and distribution of muscle and adipose tissue which can be used in the diagnosis of sarcopenia and obesity, the stratification of cardiovascular risk and the estimation of mortality rate. New tools and algorithms developing this technique and combining the DXA derivatives with other bio- or functional markers can bring the sensitivity and specificity of body composition assessment up to the level of cross-sectional techniques.

## Figures and Tables

**Table 1:** The main osteoporosis risk factors [10,11,44,45,46,47,48].

Age * Sex (female > male) * Low body mass index (BMI < 18 kg/m²) * Family history of osteoporosis * Parental hip fracture history Previous fragile fractures * Height loss (>4 cm) Excessive alcohol intake (more than two units of alcohol per day) * Smoking * Glucocorticoid intake (2.5–7.5 mg/day prednisolone administered daily or an equivalent administered for 3 months or more) * Rheumatoid arthritis * Other causes of secondary osteoporosis * (e.g., hyperparathyroidism, hypogonadism, untreated hyperthyroidism, thyroid hormone suppressive therapy, diabetes, chronic kidney disease, inflammatory bowel disease, chronic obstructive pulmonary disease) Low dietary calcium intake Vitamin D deficiency Inactivity Prolonged immobilization Susceptibility to falls

Note. * included in FRAX.

**Table 2:** Obesity categories according to DXA-based fat mass index obtained from the reference value of the NHANES [81].

		Female	Male
Fat Mass Index (kg/m²)	Normal	5–9	3–6
	Overweight	>9–13	>6–9
	Obesity Class I	>13–17	>9–12
	Obesity Class II	>17–21	>12–15
	Obesity Class III	>21	>15

## Data Availability

Not applicable.
